# Efficacy and safety of pinaverium bromide as an add‐on therapy in refractory dyspepsia: A randomized controlled trial

**DOI:** 10.1002/jgh3.13051

**Published:** 2024-03-14

**Authors:** Thansita Kamolsripat, Nithi Thinrungroj, Kanokwan Pinyopornpanish, Phuripong Kijdamrongthum, Apinya Leerapun, Taned Chitapanarux, Satawat Thongsawat, Ong‐Ard Praisontarangkul, Suwalee Pojchamarnwiputh

**Affiliations:** ^1^ Division of Gastroenterology, Department of Internal Medicine, Faculty of Medicine Chiang Mai University Chiang Mai Thailand; ^2^ Department of Radiology, Faculty of Medicine Chiang Mai University Chiang Mai Thailand

**Keywords:** antispasmodic, functional dyspepsia, omeprazole, pinaverium, randomized Controlled Trail

## Abstract

**Background and Aim:**

Functional dyspepsia (FD) remains a therapeutic challenge, and the efficacy of antispasmodic agents as adjunctive therapy is not well established. This study aimed to evaluate the efficacy and safety of pinaverium bromide added to omeprazole in treating refractory FD.

**Methods:**

We conducted a randomized, placebo‐controlled trial in patients with refractory dyspepsia. Participants were randomly assigned to receive pinaverium (50 mg, 3 times/day, n = 36) or placebo (*n* = 36) in addition to omeprazole for 8 weeks. The primary endpoint was the responder rate for adequate relief. Secondary outcomes included the Global Overall Symptom Scale (GOSS), quality of life, and safety profile.

**Results:**

No statistically significant differences were observed in the adequate relief response rate between the pinaverium bromide and control group at week 2 (58.3% vs. 62.9%, P = 0.697), week 4 (62.9% vs. 78.1%, *P* = 0.173), week 6 (64.7% vs. 75.0%, *P* = 0.363), and week 8 (64.7% vs. 75.0%, *P* = 0.363). Additionally, there were no significant differences observed in the decline of symptom score between the two groups at week 4 (8.4 ± 7.6 vs. 7.7 ± 7.1, *P* = 0.702) and week 8 (10.9 ± 8.2 vs. 8.4 ± 7.2, *P* = 0.196). Similarly, there were no significant differences in terms of quality of life between the two groups. Adverse event rates were also comparable between the two groups.

**Conclusion:**

Pinaverium bromide was found to be safe in the treatment of refractory dyspepsia, but it did not demonstrate a significant benefit in improving symptoms.

## Graphical abstract

This randomized, placebo‐controlled study investigated the addition of pinaverium bromide to omeprazole for treating refractory functional dyspepsia (FD). Results showed no significant difference between pinaverium bromide and placebo in terms of achieving adequate symptom relief, overall symptom scores, or quality of life improvements. While pinaverium bromide was well‐tolerated, the study suggests it provides no additional benefit for managing refractory FD when combined with omeprazole.

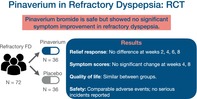

## Introduction

Functional dyspepsia (FD) is a prevalent gastrointestinal disorder with a worldwide prevalence of 6.9% by ROME IV criteria.^1^ The condition is characterized by two subtypes: postprandial distress syndrome (PDS) and epigastric pain syndrome (EPS), which may overlap.^2^ Despite its benign nature, FD often causes unpleasant symptoms that affect patients' physical, psychological, social, and economic well‐being.^3^

Proton pump inhibitors (PPIs) are currently considered first‐line therapy according to current guidelines.^4^, ^5^ However, their response rate varies between 30% and 60%, with minimal therapeutic benefit over placebo (10%–20%).^6^, ^7^, ^8^, ^9^ Despite the availability of new treatment options with alternative mechanisms of action, the evidence supporting their use remains limited.

Duodenal low‐grade inflammation has been proposed as a potential pathogenesis of FD. Several studies have demonstrated an increase in eosinophils and mast cells in the duodenal tissue of FD patients.^10^, ^11^ This inflammation may lead to decreased mucosal integrity and abnormal smooth muscle contractions.^12^, ^13^ Previous research from our group has shown that a combination of antispasmodic and anxiolytic drugs (clidinium bromide/chlordiazepoxide) improves symptoms and quality of life in FD patients refractory to PPIs.^14^ However, the effect of antispasmodic monotherapy as an add‐on treatment to PPIs in FD remains undefined.

Pinaverium bromide is an antispasmodic drug acting on a muscarinic receptor with high safety profile. We hypothesize that adding pinaverium bromide to a PPI might assist the treatment in PPI‐resistant dyspepsia. Therefore, we conducted a randomized, double‐blind, placebo‐controlled trial aimed to evaluate the efficacy and safety profile of pinaverium bromide add‐on to PPI in FD patients who had incomplete response to PPI therapy.

## Methods

### Study design

This study was a single‐center, double‐blind, randomized controlled trial carried out at Chiang Mai University Hospital from June 2021 to August 2022, in accordance with the Declaration of Helsinki and Good Clinical Practice guidelines. All patients provided written informed consent prior to participation. The study was approved by the Institutional Review Board (MED‐2563‐07700) and registered with the Thai Clinical Trials Registry (TCTR20210819002).

### Participants

We included patients aged 18–70 years who met the Rome IV criteria for a diagnosis of FD and had persistent symptoms despite receiving a standard dose of PPIs for at least 8 weeks before enrollment. Additionally, all patients had normal results from upper endoscopy and tested negative for Helicobacter pylori infection within the 3 years prior to enrollment.

We excluded patients who met any of the following criteria: (a) concurrent diagnosis of gastroesophageal reflux disease (GERD), irritable bowel syndrome (IBS), or defecation problems; (b) presence of severe comorbidities or end‐stage renal disease; (c) pregnancy or breastfeeding or had planned pregnancies; (d) known history of allergies to pinaverium bromide or PPIs. Additionally, any patient currently taking nonsteroidal anti‐inflammatory drugs was also excluded from the study.

### Randomization and intervention

Computer‐generated blocking randomization was used for patients' randomization to receive either pinaverium or placebo add‐on to standard dose omeprazole. An independent staff member assigned the treatments according to consecutive numbers, which were kept in sealed envelopes. All investigators and patients were blinded to treatment allocation.

Eligible patients had a 2‐week drug washout period including PPIs, prokinetics, simethicone, alginate, and antispasmodic drugs before starting the study medication. Then, patients were randomly assigned to receive either pinaverium bromide (Dicetel®, Abbott Inc.) (50 mg, 3 times/day) or placebo (identical starch‐containing capsule) add‐on to standard dose omeprazole (20 mg once daily) for 8 weeks. During the study, the participants were advised to avoid using over‐the‐counter medication. An antacid was given as rescue medicine. Compliance was checked by pill count at week 4 and week 8.

### Data collection and outcome assessment

#### Data collection

Baseline characteristics were recorded including age, gender, medical illness, psychiatric condition, FD subtype by Rome IV criteria, the Hospital Anxiety and Depression Scale (HADS),^15^ FD specific Global Overall Symptom Scale (GOSS), and quality of life.

Self‐report of adequate relief was recorded in the given diary; patients were noted whether they were satisfied with the symptom relief or not by answering ‘yes or no’ every day in their diary during 8‐week study period. Patients were considered as a responder if they reported adequate relief over 50% of days during the period of treatment. Dyspeptic symptom score was assessed using a GOSS^16^ (using the 7‐point Likert dyspepsia severity scale) every 4 weeks. The quality of life was assessed using the short‐form Nepean Dyspepsia Index^17^ at baseline and post‐treatment. The translation of questionnaire had undergone the forward–backward translation method by the language institute Chiang Mai University and assessed by three independent gastroenterologists for content validity and clarification of the language. The short‐form Nepean Dyspepsia Index comprises of five subcategories: interference, knowledge/control, eating/drinking, sleep disturbance, work/study, and scores, summarized into an overall quality of life score ranging from 10 to 50. Higher scores on the GOSS and short‐form Nepean Dyspepsia Index indicated more severe symptoms and a lower quality of life, respectively.

#### Outcomes and definitions

The primary outcome, the adequate relief response rate, was defined as the proportion of patients reporting satisfactory symptom relief on more than 50% of days during the treatment period. Secondary outcomes included improvement in quality of life as measured by the short‐form Nepean Dyspepsia Index, changes in overall symptom scores on the GOSS, and the safety profile assessed through the incidence of adverse events.

#### Statistical analysis

According to previous research,^18^ the power calculation assumed of 40% responder in the placebo arm. As this was the first study of pinaverium bromide in FD patients, based on therapeutic gain of the clidinium/chlordiazepoxide in FD,^14^ we expect a 35% therapeutic gain over placebo. The sample size calculation used an estimate of 40% responders in the placebo group and 75% responders in the treatment group with a 5% α‐error and a 20% β‐error; assuming a 20% drop‐out rate, the trial required at least 36 patients in each arm.

The primary outcome was analyzed based on the intention‐to‐treat analysis. Chi‐square test or Fisher's exact test was used to evaluate the difference between the two treatment arms in terms of the proportion of responders, and the rates of adverse events. The dyspepsia symptom score and quality of life scores were compared between the two treatment arms using an independent t‐test or Mann–Whitney U test as appropriate. All statistical analyses were performed using Stata (version 16.0 for Windows, StataCorp LCC). A *P*‐value less than 0.05 was considered a statistically significant difference.

## Results

### Baseline characteristics

**Figure 1:**
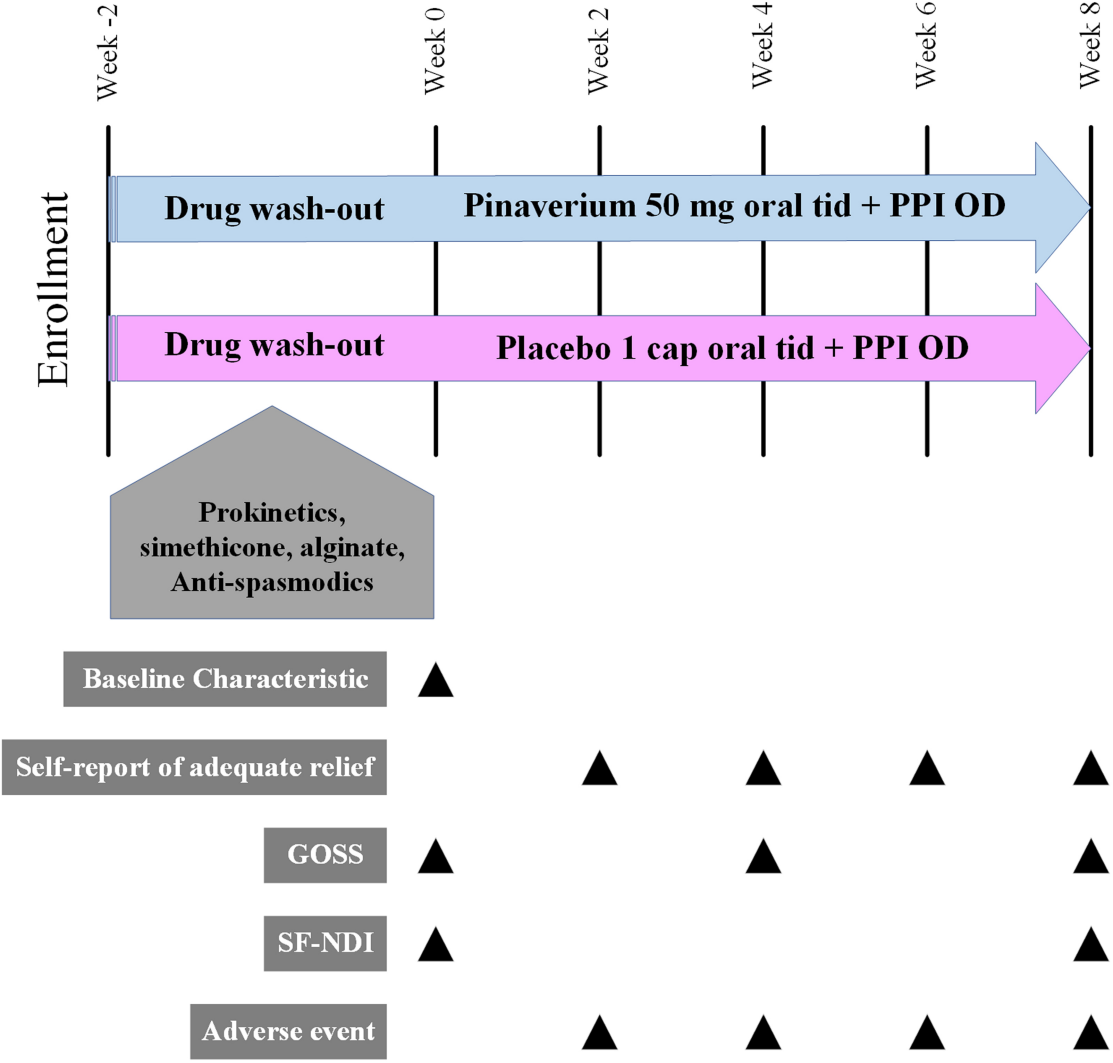
Study design. GOSS, Global Overall Symptom Scale; SF‐NDI, short‐form Nepean Dyspepsia Index.

One‐hundred and seven outpatients with the diagnosis of FD were screened. Of these, 35 patients were excluded. The remaining 72 patients were randomly assigned to receive pinaverium bromide (36 patients) or placebo (36 patients) (Fig. 1). The baseline characteristics of patients are shown in Table 1. The mean age was 51 years. Fifty‐five patients (76.4%) were female. Overall, 19 patients (26.4%) had PDS, 17 patients (23.6%) had EPS, and 36 patients (50.0%) had overlap syndrome. The median duration of symptoms prior to study participation was 15 months (range, 6–36 months). The mean baseline short‐form Nepean Dyspepsia Index (SF‐NDI) was 32.0 ± 9.1 and the mean GOSS was 25.1 ± 6.5.

**Table 1:** Baseline characteristics of the study populations (*N* = 72)

Characteristic	Total (*N* = 72)	Pinaverium (*N* = 36)	Control (*N* = 36)	*P*‐Value
Sex, *n* (%)				0.094^†^
Male	17 (23.6)	5 (13.9)	12 (33.3)	
Female	55 (76.4)	31 (86.1)	24 (66.7)	
Age, mean ± SD	51.7 ± 13.3	51.9 ± 11.1	51.4 ± 15.4	0.861^‡^
BMI, mean ± SD	23.3 ± 3.0	24.1 ± 3.0	22.4 ± 2.9	0.012^‡^
FD types, *n* (%)				0.614^§^
Overlap	36 (50.0)	16 (44.4)	20 (55.6)	
Epigastric pain syndrome	17 (23.6)	9 (25.0)	8 (22.2)	
Postprandial distress syndrome	19 (26.4)	11 (30.6)	8 (22.2)	
Duration of symptoms, Median (IQR)	15 (6–36)	21 (6.5–36)	12 (6–36)	0.638^¶^
Underlying disease, *n* (%)				
Hypertension	13 (18.1)	6 (16.7)	7 (19.4)	0.759^‡^
Diabetes	6 (8.3)	1 (2.8)	5 (13.9)	0.199^†^
Chronic kidney diseases	1 (1.4)	1 (0.2)	0 (0)	1.000^†^
Cirrhosis	2 (2.78)	1 (2.78)	1 (2.78)	1.000^†^
Others	20 (27.8)	9 (25.0)	11 (30.6)	0.599^§^
HADS anxiety, Median (IQR)	4 (2–7)	4 (1–7)	4 (2.5–7.5)	0.905^¶^
HADS depression, Median (IQR)	0 (0–4)	0.5 (0–3.5)	0 (0–4.5)	0.812^¶^
Short‐from Nepean Dyspepsia Index, Mean ± SD	32.0 ± 9.1	31.7 ± 9.9	32.4 ± 8.2	0.747^‡^
Global overall symptoms scale, Mean ± SD	25.1 ± 6.5	25.0 ± 7.0	25.1 ± 6.1	0.972^‡^
Missed pill during intervention, *n* (%)	6 (8.33)	3 (8.33)	3 (8.33)	1.000^†^

^†^ Fisher's exact test.

^‡^ Student's *t*‐test.

^§^ Chi‐square test.

^¶^ Mann–Whitney–Wilcoxon test or the Wilcoxon rank sum test.

Abbreviation: IQR, interquartile range.

**Figure 2:**
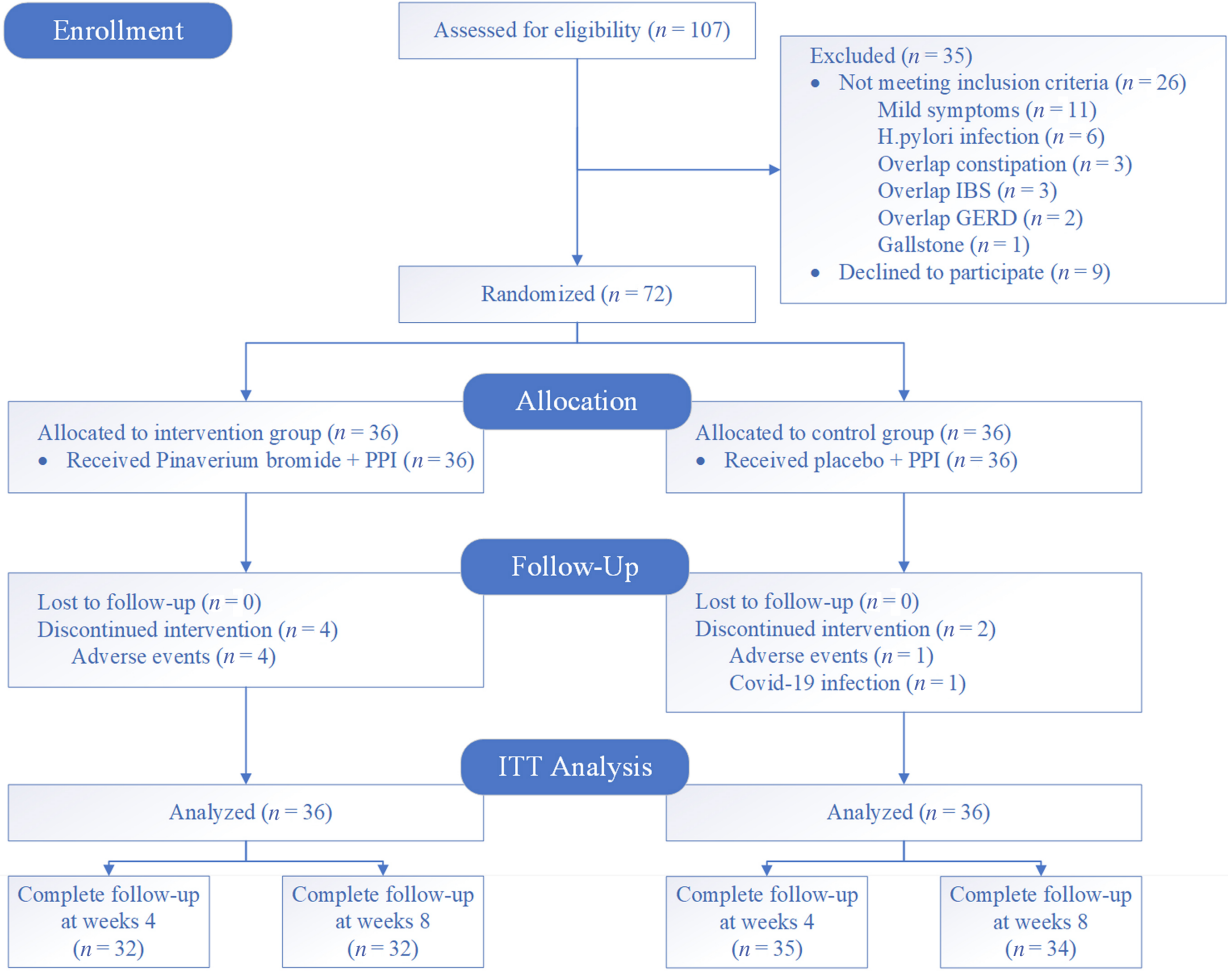
Consort flow diaphragm of study protocol. GERD, gastroesophageal reflux disease; IBS, irritable bowel syndrome.

The baseline characteristics of the two groups were comparable, except for BMI, which was slightly higher in the pinaverium group (mean of 24.1 ± 3.0 vs. 22.4 ± 2.9, *P* = 0.012). Six patients (8.3%) dropped out of the study, four from the pinaverium group, and two from control group. A total of 66 patients completed the trial, 32 in the treatment group and 34 in the control group (Fig. 2). The overall compliance was greater than 90% for all participants.

### Self‐report of adequate relief

**Figure 3:**
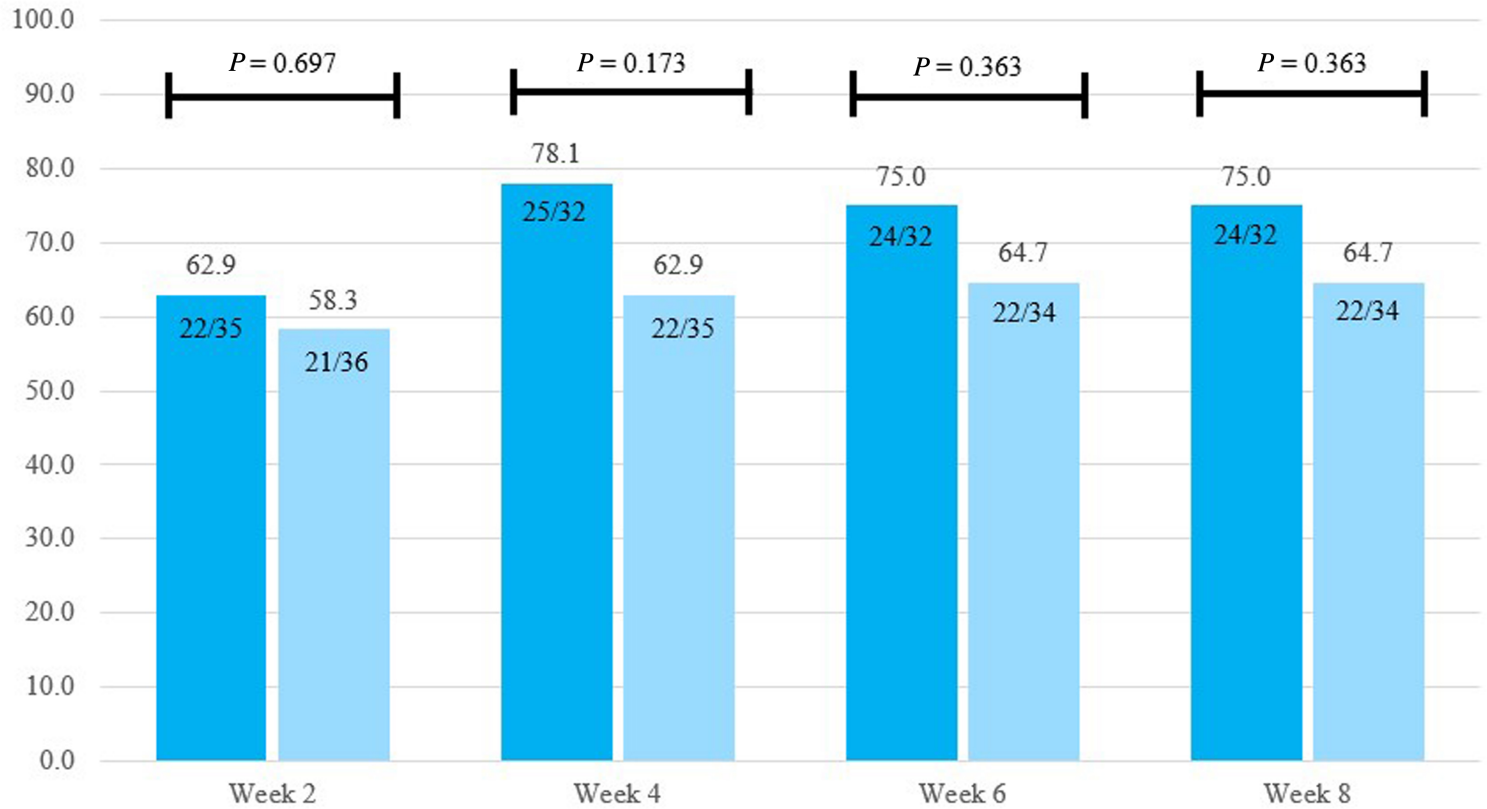
Rate of responders (self‐report of adequate relief, %) between groups by intention‐to‐treat analysis. 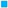 pinaverium; 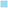, control.

According to ITT analysis, there was no significant difference in the primary outcome, rate of responders, between the pinaverium group and the placebo group at various time points throughout the 8‐week treatment period (58.2% vs. 62.9% at week 2, 62.9% vs. 78.1% at week 4, 64.7% vs. 75.0% at week 6, and 64.7% vs. 75.0% at week 8) (Fig. 3). No notable variations were observed when results were analyzed using per‐protocol analysis.

### Dyspepsia symptom score (seven‐point GOSS)

**Figure 4:**
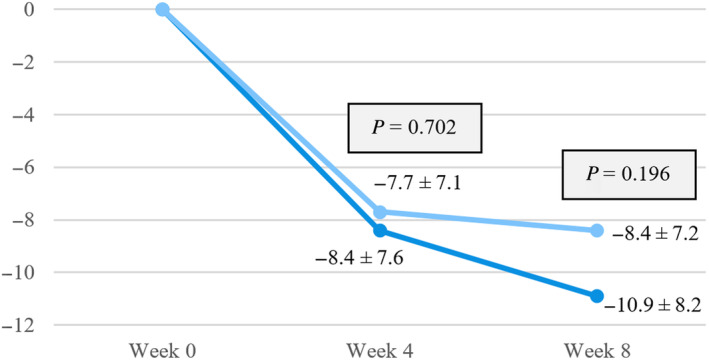
Mean difference in global overall symptom scale between groups. 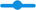 pinaverium; 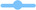, placebo.

There was no difference in the change of total global overall symptom score between the pinaverium group and the placebo group at week 4 (−8.4 ± 7.6+ vs. −7.7 ± 7.1, *P* = 0.702) and week 8 (−10.9 ± 8.2 vs. −8.4 ± 7.2, *P* = 0.196) (Fig. 4). After symptom subtype analysis, only early satiety symptom was significantly improved in the pinaverium group compared with the placebo group (−2.1 ± 2.2 and−0.7 ± 2.0, *P* = 0.012), but there were no significant differences between the two groups for other symptom subtypes, including epigastric pain, excessive belching, epigastric burning, abdominal bloating, and postprandial fullness. (Table 2).

**Table 2:** Secondary outcome showing seven‐point global overall symptom scale between groups, mean ± SD

	Pinaverium (*N* = 32)			Placebo (*N* = 34)			
Modalities	Week 0	Week 8	Change	Week 0	Week 8	Change	*P*‐Value
Total scores	24.4 ± 6.9	13.5 ± 5.6	−10.9 ± 8.2	24.5 ± 5.8	16.1 ± 6.4	−8.4 ± 7.2	0.196^†^
Epigastric pain	5.0 ± 1.4	2.8 ± 1.7	−2.2 ± 2.1	4.8 ± 1.4	3.0 ± 1.6	−1.8 ± 2.0	0.471^†^
Excessive Belching	3.1 ± 1.8	2.0 ± 1.4	−1.2 ± 1.9	3.6 ± 1.8	2.3 ± 1.4	−1.4 ± 1.8	0.621^†^
Epigastric burning	3.6 ± 2.0	2.1 ± 1.2	−1.6 ± 2.0	3.4 ± 1.6	2.3 ± 1.6	−1.1 ± 1.9	0.332^†^
Abdominal bloating	4.6 ± 1.7	2.9 ± 1.6	−1.7 ± 2.1	4.9 ± 2.0	2.9 ± 1.6	−1.9 ± 2.1	0.591^†^
Postprandial fullness	4.3 ± 2.2	2.0 ± 1.4	−2.3 ± 2.2	4.3 ± 1.7	2.8 ± 1.6	−1.5 ± 2.0	0.131^†^
Early satiety	3.8 ± 2.2	1.8 ± 1.1	−2.1 ± 2.2	3.5 ± 1.9	2.8 ± 1.8	−0.7 ± 2.0	0.012^†^

^†^ Student's *t*‐test.

### Quality of life

Baseline SF‐NDI scores for overall quality of life and the five subscales were comparable between two groups. After 8 weeks of treatment, there was no significant difference in the decrease of SF‐NDI scores between the two groups. (Table 3).

**Table 3:** Secondary outcome showing the short‐form Nepean Dyspepsia Index between groups, mean ± SD

	Pinaverium (*N* = 32)			Placebo (*N* = 34)			
Modalities	Week 0	Week 8	Change	Week 0	Week 8	Change	*P*‐Value
Overall	30.6 ± 9.8	19.5 ± 9.1	11.1 ± 10.8	32.2 ± 8.2	20.1 ± 7.8	12.1 ± 9.5	0.683^†^
Tension	7.0 ± 2.3	4.6 ± 2.3	2.3 ± 2.9	7.4 ± 2.0	4.4 ± 2.3	3.1 ± 2.8	0.239^†^
Interference with daily activities	6.3 ± 2.5	3.8 ± 2.3	2.2 ± 2.6	5.7 ± 2.7	3.5 ± 1.8	2.3 ± 2.7	0.870^†^
Eating/drinking	5.9 ± 3.0	3.6 ± 2.1	2.0 ± 3.4	6.6 ± 2.7	4.2 ± 2.5	2.4 ± 2.9	0.680^†^
Knowledge/control	6.3 ± 2.8	3.4 ± 2.1	2.7 ± 2.9	6.9 ± 3.0	4.4 ± 2.7	2.3 ± 3.6	0.683^†^
Work/study	6.0 ± 3.0	4.0 ± 2.3	2.0 ± 3.3	5.8 ± 2.8	3.7 ± 1.9	2.1 ± 2.9	0.907^†^

^†^ Student's *t*‐test.

#### Safety profile

There were 14 reported adverse events in the trial, with no significant difference in rate between the pinaverium and control groups (27.8% vs. 47.2%, respectively; *P* = 0.088). The most common side effects were dizziness and epigastric burning (Table 4). Four patients in pinaverium group did not complete the study due to adverse events (two epigastric burning, two abdominal pain). There were no serious adverse events during the 8 weeks of study.

**Table 4:** Adverse event between groups

Adverse event	Total (*N* = 72)	Pinaverium (*N* = 36)	Control (*N* = 36)	*P*‐Value
Chest pain	3 (4.2)	3 (8.3)	0 (0)	0.239^†^
Odynophagia	1 (1.4)	1 (2.8)	0 (0)	1.000^†^
Abdominal pain	5 (6.9)	4 (11.1)	1 (2.8)	0.357^†^
Diarrhea	1 (1.4)	0 (0)	1 (2.8)	1.000^†^
Nausea	2 (2.8)	1 (2.8)	1 (2.8)	1.000^†^
Vomiting	4 (5.6)	3 (8.3)	1 (2.8)	0.614^†^
Pruritus	1 (1.4)	0 (0)	1 (2.8)	1.000^†^
Dizziness	7 (9.7)	4 (11.1)	3 (8.3)	1.000^†^
Constipation	4 (5.6)	4 (11.1)	0 (0)	0.115^†^
Others (*N* = 12)	12 (16.7)	8 (22.2)	4 (11.1)	0.343^†^
Epigastric burning	6 (50.0)	5 (62.5)	1 (25.0)	0.221^§^
Palpitation	2 (16.7)	1 (12.5)	1 (25.0)	1.000^†^
Weight gain	1 (8.3)	1 (12.5)	0 (0)	1.000^†^
Bloating	1 (8.3)	1 (12.5)	0 (0)	1.000^†^
Excessive passage of gas	1 (8.3)	1 (12.5)	0 (0)	1.000^†^

^†^ Fisher's exact test.

^§^ Chi‐square test.

## Discussion

This randomized placebo‐controlled trial aimed to assess the efficacy and safety of pinaverium bromide as an add‐on to omeprazole for the treatment of refractory dyspepsia. The study found no statistically significant differences in the primary endpoint of adequate relief response rate between the pinaverium bromide and placebo groups. Additionally, there were no significant differences observed in the secondary outcomes, such as the Global Overall Symptom Scale (GOSS) and quality of life, between the two groups.

In this study, the responder rate in the control group was found to be around 60%, which is higher than the placebo response rate reported in other studies^19^, ^20^ and our previous research.^14^, ^19^ This high placebo response rate may be attributed to the effect of omeprazole given in the control group. Even though we only enrolled PPI nonresponsive patients, some participants may have previously received non‐omeprazole PPI, which could have caused a PPI switching effect. Additionally, the discontinuation of PPI during the washout period and the re‐introduction of PPI may have also improved the participants' symptoms. Furthermore, all participants underwent upper endoscopy and ultrasonography before enrollment, which may have reduced their stress and anxiety, leading to an increased placebo response rate in the study.

Clidinium/Chlordiazepoxide, a combination of antispasmodic and anxiolytic drugs, has been shown to be beneficial in dyspepsia in our previous study.^14^ However, in this study, pinaverium bromide, a pure anti‐spasmodic drug, failed to show the same results. Our results suggest that the patients in the clidinium/chlordiazepoxide study might respond mainly on the effect of anxiolytic effect over antispasmodic effect.

FD is composed of complex pathophysiology. Our hypothesis of interest was duodenal low‐grade inflammation causing abnormal contraction of intestinal smooth muscles resulting in dyspeptic symptoms.^10^, ^11^, ^12^, ^13^ Our results suggest that the modulation of abnormal intestinal contraction may not be beneficial for all patients with FD. However, subtype analysis revealed improvement in early satiety, a common symptom of PDS. It is known that duodenal inflammation is prominent in FD patients with post‐prandial distress syndrome.^21^ Therefore, further research is needed to explore the potential benefits of antispasmodics in this specific subgroup of FD patients.

There are several strengths of our study. To the best of our knowledge, this is the first randomized controlled trial to study the effect of anti‐spasmodic in refractory FD patients. Strict inclusion criteria were applied, including Rome IV criteria and significant symptoms impacting quality of life. Both upper endoscopy and upper abdominal ultrasonography were conducted to exclude organic diseases. Additionally, strict exclusion criteria were applied to minimize overlap with other functional gastrointestinal disorders, specifically IBS, for precise outcome assessment.

Our study has several limitations that must be taken into account when interpreting the results. The first limitation is the potential lack of generalizability due to the study's single‐center, university referral hospital setting. Additionally, the small sample size may have resulted in the study being underpowered to detect a real treatment effect. This should be considered when interpreting the results and interpreting the lack of significant differences in the primary and secondary outcomes. Furthermore, the study design did not include an assessment of gastrointestinal motility via physiological testing or duodenal biopsy to evaluate for the presence of inflammation, which could have further provided more insights into the results.

Despite these limitations, the study still provides important information on the safety profile of pinaverium bromide in this population and can serve as a basis for future research. Future studies should consider increasing the sample size and incorporating these assessments in order to enhance the power of the study and better understand the potential benefit of pinaverium bromide in the treatment of refractory dyspepsia.

In conclusion, our study found no significant benefit of adding pinaverium bromide, an anti‐spasmodic drug, to PPI for treating refractory FD. Despite these findings, the drug demonstrated a good safety profile with no serious adverse effects. Further research with larger sample sizes is necessary to confirm these findings and explore potential benefits in specific subgroups.
